# Nervous Headache

**Published:** 1844-07

**Authors:** 


*Nervous Headache.*—Dr. R. Howard prescribes, with success, for nervous and various other headaches, a mixture of a drachm of acetic acid, an ounce of compound tincture of cardamoms, and four ounces of some convenient vehicle, of which he directs a mouthful to be taken every 20 minutes.

*London Lancet.*

